# Notes and News

**Published:** 1901-01-05

**Authors:** 


### Notes and News.

The plague is still prevalent in Mauritius, the deaths averaging upwards of thirty per week.

Grants made by the Leathersellers Company during 1900 from their Corporate Funds included £4,844 to hospitals and other charitable institutions and patriotic funds.

In commenting on the enormous waste caused by alcoholism, the Paris *Journal de Médecine* points out that hospital statistics alone witness that inveterate drunkenness costs the city at least 2,000,000 fr. per annum, through the lost labour of the individual and the direct cost of hospital treatment.

Addenbrooke's Hospital, Cambridge, has again become the recipient of a munificent gift of £1,000 from the Chairman, Mr. Alexander Peckover, Lord-Lieutenant of the County. This gift is to mark the commencement of the new century, and to free the accounts of the hospital from the arrears against it.

Amongst the list of New Year honours we find the names of the following medical men. Dr. Selby Church, President of the Royal College of Physicians, and Dr. Thomas Barlow, physician to the Queen's household, have been created baronets. Dr. Hugh Adcock, physician to the Shah of Persia, has been knighted; Sir William Turner, D.C.L., has received the K.C.B., and Dr. Frank Simon, Principal Medical Officer to the Straits Settlements, has been made Companion of the Order of St. Michael and St. George.

The Anthropological Institute proposes to publish a periodical to be entitled *Man.* It is to be a monthly record of progress in the various branches of the study of man, these various branches of study being treated more fully in proportion as they are less adequately provided for by existing periodicals. This is a laudable effort to fill up a gap, or rather a whole row of gaps, in scientific journalism, and we wish it every success. Like other things, however, gaps have a cause, and in journalism this cause is usually a lack of general interest in the topic in question. The editor, then, who sets to work to run even a monthly paper on topics which other editors have not cared to deal with, undertakes a very difficult task. He has our sympathy.

An Infectious Diseases Hospital is to be erected for the joint use of the burghs of Banff, Macduff, Portsoy, Cullen, and Buckie in Scotland. Mr. James Campbell of Cullen has generously assisted the scheme by a gift of £3,000.

The books of the Hospital Saturday Fund will close on January 7th, when it is hoped that the sum realised last year, namely, £20,000, will be reached on the present occasion. Up to November 24th the collection was less than that of the previous year

At Newton Abbot a druggist was recently censured for prescribing a medicine containing rhubarb and magnesia for a child whom he had not seen. Eventually medical advice was obtained, when the child was found to be suffering from acute peritonitis, from which it died.

Lord Powerscourt writes to the *Times* suggesting that every town or district should levy a hospital rate. Lord Powerscourt does not explain in his letter whether this rate is to support or subsidise the hospitals. If the first, it is a proposal that the hospitals should be rate-supported. He does not say in what way this would affect the Poor Law institutions, for which the community are already paying; in fact, he leaves the bare suggestion for others to consider in a practical form.

A very active agitation for the provision of "Kosher" food for the Jewish poor when inmates of the hospitals is taking place in Manchester. Those taking part in it are rather divided on the subject of the direction which this provision should take. On one hand the authorities of the hospitals have been approached with a view to setting apart a ward for Jews, others desire the establishment of a Jewish hospital, and a third party is in favour of an outside "Kosher" kitchen to supply the Jewish patients with food. The London Hospital offers an object lesson to the hospitals which might set apart a ward. The Jewish ward in that institution has been a complete success. The establishment of a separate hospital and its maintenance would be infinitely more costly than the complete support of a ward and its attendant kitchen in an already existing hospital. The proposal to supply patients from an outside kitchen is quite impracticable.

A plan for the reconstruction of the Glasgow Royal Infirmary has been adopted by the committee. The plan shows a quadrangle with a central tower in the frontage towards Cathedral Square. At the angles are circular turrets. The building will be divided into three sections—medical, surgical, and special diseases. The front or Jubilee Block is six storeys in height, and contains ten large wards and twenty single ones, accommodating 225 patients. The surgical block, also six storeys high, contains eighteen large and twenty-four small wards, with accommodation for 296 patients. The special diseases block, in addition to its own proper wards, contains the administration. The kitchens are on the top floor, whence a service corridor extends throughout the building, and is supplied with service lifts. Every block contains dwelling accommodation for a resident surgeon. A fine operating theatre is provided, with sterilising and other necessary rooms adjoining. The roof of the central block is flat, and will be utilised as a garden. The ventilation will be by artificial means. The hospital, which will accommodate about 700 patients, is planned by Mr. Miller.

